# Impact of prelacteal feeds and neonatal introduction of breast milk substitutes on breastfeeding outcomes: A systematic review and meta‐analysis

**DOI:** 10.1111/mcn.13368

**Published:** 2022-04-30

**Authors:** Rafael Pérez‐Escamilla, Amber Hromi‐Fiedler, Elizabeth C. Rhodes, Paulo A. R. Neves, Juliana Vaz, Mireya Vilar‐Compte, Sofia Segura‐Pérez, Kate Nyhan

**Affiliations:** ^1^ Department of Social and Behavioral Sciences Yale School of Public Health New Haven Connecticut USA; ^2^ International Center for Equity in Health Universidade Federal de Pelotas Pelotas Rio Grande do Sul Brazil; ^3^ Faculty of Nutrition Universidade Federal de Pelotas Pelotas Rio Grande do Sul Brazil; ^4^ Department of Public Health Montclair State University Montclair New Jersey USA; ^5^ Chief Progam Officer Hispanic Health Council Hartford Connecticut USA; ^6^ Harvey Cushing/John Hay Whitney Medical Library Yale University New Haven Connecticut USA

**Keywords:** breastfeeding, breast milk substitutes, infant feeding, meta‐analysis, neonatal period, prelacteal feeds

## Abstract

The introduction of fluids other than breast milk during the first few days of life or later neonatal period has been identified as a risk factor for suboptimal breastfeeding (BF) outcomes in numerous studies using varying study designs. However, the relationship between early introduction of fluids other than breast milk and BF outcomes has not been systematically assessed using only prospective studies that can establish temporality, which is critical for determining whether observed associations are causal. We conducted a systematic review and meta‐analysis of prospective studies to assess if there is a difference in BF outcomes as a result of the introduction of: (a) milk‐based prelacteals, (b) water‐based prelacteals and (c) breast milk substitutes (BMS) between 4 days and 4 weeks postpartum. We searched PubMed, Lilacs, Web of Science and other repositories for original research investigating the relationship between early introduction of prelacteals and/or BMS and BF outcomes. Forty‐eight studies met the inclusion criteria for the systematic review. Of the 39 prelacteal feeding studies, 27 had the prerequisite statistical information for inclusion in the meta‐analysis. Findings from the meta‐analysis showed a relationship between prelacteals and exclusive BF cessation (RR 1.44; 1.29–1.60) and any BF cessation (2.23; 1.63–3.06) among infants under 6 months old. Nine studies focusing on the introduction of BMS during the neonatal period identified this practice as a statistically significant risk factor for a shorter BF duration. Effective interventions are needed to prevent the introduction of unnecessary milk‐based prelacteals and BMS during the perinatal and neonatal periods to improve BF outcomes.

## Key messages

- The practice of prelacteal feeding and introduction of breastmilk substitutes (BMS) during the neonatal period is widespread across the globe.
- In this systematic review and meta‐analysis of prospective studies, the introduction of prelacteals was associated with suboptimal breastfeeding practices including a higher risk of not exclusive breastfeeding and any breastfeeding cessation among infants under 6 months of age.
- Prospective studies show that the introduction of BMS during the neonatal period is a risk factor for suboptimal breastfeeding practices.
- Facility and community‐based interventions are needed to prevent the introduction of prelacteals perinatally and BMS during the neonatal period.

## Graphical abstract

Findings from the meta‐analysis showed a relationship between prelacteals and lack of exclusive breastfeeding and any breastfeeding cessation among infants under 6 months old. Effective interventions are needed to prevent the introduction of unnecessary milk‐based prelacteals during the perinatal and neonatal periods to improve breastfeeding outcomes.

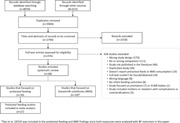

## 1 INTRODUCTION

Timely initiation of breastfeeding (BF) and preventing the unnecessary introduction of prelacteal feeds, defined as fluids other than breast milk offered during the first 3 days after birth and breast milk substitutes (BMS) during the neonatal period is key for the subsequent success of BF and the reduction of neonatal mortality (Boccolini et al., 2013). For example, prelacteal feeding and early introduction of BMS have been consistently associated with suboptimal BF practices, such as delayed initiation of BF, shorter exclusive breastfeeding (EBF) and any BF durations, mainly through ecological, cross‐sectional or retrospective studies (Boccolini et al., 2015; Neves et al., 2022; Pérez‐Escamilla et al., 1996; Segura‐Pérez et al., 2022) and infant morbidity and mortality (Nguyen et al., 2020). Yet, about one in three children in low‐ and middle‐income countries (LMICs) receive unnecessary milk‐ and/or water‐based prelacteal feeds (Neves et al., 2022).

A recent analysis of 76 LMICs (Neves et al., 2022) found that milk‐based prelacteals were more common than water‐based prelacteals in higher‐income countries while the opposite was true in lower‐income countries. By contrast, water‐based prelacteals were relatively more common in lower‐income countries. More specifically, milk‐based prelacteals were most commonly used in Eastern Europe and Central Asia, East Asia and Pacific and in Latin America and Caribbean regions. Yet, in the 3 African regions, water‐based prelacteal foods were the most prevalent although milk‐based prelacteals were also used. In addition to the introduction of prelacteals during the first 3 days of life, BMS are commonly introduced during the neonatal period across the globe (Neves et al., 2022).

Given that ecological, cross‐sectional and retrospective studies can lead to spurious associations because of recall bias, it is key to find out if there is a relationship between prelacteals and BF outcomes focusing only on prospective studies. This study needs to address the use of milk‐ and water‐based prelacteals together and separately as there are contrasting regional prelacteal feeding patterns across the globe (Neves et al., 2022). Therefore, the objectives of this study, as listed in the original protocol, were to conduct a systematic review and meta‐analysis of prospective studies to answer the following questions: (a) Is there a difference in BF outcomes when milk‐based prelacteal feeds are introduced compared to when they are not? (b) Is there a difference in BF outcomes when water‐based prelacteal feeds are introduced compared to when they are not? and (c) Is there a difference in BF outcomes when BMS are introduced between 4 days and 2 weeks and >2 weeks and 4 weeks postpartum compared to when they are not? We hypothesize that prelacteal feeds may undermine BF success by delaying BF initiation, reducing nursing frequency, delaying the onset of lactation, increasing the risk of very early introduction of BMS, reducing milk supply and increasing the frequency of BMS feeding beyond the neonatal period further undermining breast milk production (Pérez‐Escamilla et al., 2019).

Given that previous studies have already identified modifiable risk factors for prelacteal feeding and introduction of BMS during the neonatal period (Akuse & Obinya, 2002; Boccolini et al., 2015; Kavle et al., 2017; Neves et al., 2022; Segura‐Pérez et al., 2022), we anticipated that the findings from this review could help advance infant feeding and maternity care policies that are more supportive of BF, especially during the crucial period when the milk supply starts to get established (Boss et al., 2018).

## 2 METHODS

This systematic review and meta‐analysis followed the Institute of Medicine guidelines. Before reviewing the literature, the protocol was developed and registered in PROSPERO (ID# CRD42021240669). BF outcomes of interest were grouped into short‐, medium‐ and longer‐term outcomes. The outcomes of this review were EBF duration or prevalence among infants less than 6 months old, duration/prevalence of EBF reported beyond 1 month postpartum and the duration/prevalence of any BF until 1 year postpartum. The short term outcome delayed onset of lactation, that is, milk arrival >3 days after birth, was listed in the original protocol, however, it was decided not to include it because it was an outcome in another systematic review published in this supplement (Segura‐Pérez, et al., 2022). For this review, definitions from the World Health Organization were used for the timely initiation of BF, EBF and any BF (UNICEF, 2016). *Timely initiation of BF* was defined as BF initiation within 1 h after birth. *Exclusive breastfeeding* was defined as the infant receiving only breast milk with no other liquids or solids introduced (exceptions were that the infant can receive medicines, vitamins/minerals in liquid form and oral rehydration solution). *Any breastfeeding* was defined as the infant receiving breast milk either directly from the breast or expressed breast milk.

The two exposures explored in this systematic review were prelacteals and the introduction of BMS during the neonatal period. Prelacteal feeds were defined as any fluids other than breast milk given during the first 3 days postpartum (Neves et al., 2022). Prelacteal feeds were characterized as being water‐ or milk‐based. It was not possible to classify milk‐based prelacteals further as infant formula versus other types of animal milk because this information was not available. The introduction of BMS during the neonatal period was defined as the feeding of any milk‐based BMS between 4 days and 4 weeks postpartum.

### 2.1 Search strategy

We searched the databases MEDLINE All (via Ovid), Web of Science Core Collection (as licensed at Yale University, including SCI‐EXPANDED 1900–, SSCI 1900–, A&HCI 1975–, CPCI‐S 1991–, CPCI‐SSH 1991–, BKCI‐S 2005–, BKCI‐SSH 2005–, ESCI 2015– and CCR‐EXPANDED 1985‐), PsycINFO (via Ovid), EMBASE (via Ovid), LILACS (via the Virtual Health Library Regional Portal), SciELO and Global Index Medicus databases. The searches were run between March and May 2021. We searched for articles containing one or more controlled vocabulary terms or keywords related to both of the following concepts: infant feeding outcomes and prelacteal feeds. The latter concept was operationalized with text words and adjacency statements addressing in‐hospital supplementation and early introduction of BMS, as well as prelacteals per se (Table S1). The search was designed, tested and run by a team that included public health scientists with expertise in BF research (R. P. E., A. H. F., E. C. R.) and a medical librarian (K. N.). A reproducible search strategy from all the database searches is available at https://osf.io/jkx6s/. The search results were combined and de‐duplicated in EndNote. To ensure no relevant studies were left out, we conducted backward citation chaining through the identification of studies that were cited in previously published systematic reviews, consulted with experts in the field and reviewed researchers' files to identify additional articles to be included. This use of both bibliographic databases and citation networks helped ensure the comprehensive retrieval of relevant papers.

### 2.2 Study selection criteria

The records identified from the database searches were screened using Covidence. Studies were included in this review if they: (a) were published in English, Spanish, or Portuguese; (b) used a prospective cohort, quasi‐experimental, or randomized controlled trial (RCT) design; (c) reported BF duration and other BF outcomes. Studies were excluded if they: (a) were cross‐sectional studies, case‐control studies, clinical studies, qualitative studies, reviews, systematic reviews, or meta‐analyses; (b) included women who had a delivery involving serious maternal complications such as severe post‐partum haemorrhaging that prevented them from BF; (c) included women with contraindications to BF such as cancer chemotherapy and taking lithium medications; (d) included newborns without serious medical complications such as asphyxia at birth; (e) focused on premature or very low birth weight babies; (f) did not report prelacteal feeds or early introduction of BMS; (g) did not report any longer‐term BF outcomes (i.e., duration/prevalence of any BF) and (h) were not published in the peer‐reviewed literature—for example, technical reports, dissertations, conference abstracts.

Titles and abstracts of studies were independently screened for inclusion by two reviewers (A. H. F., E. C. R.) and those that did not meet the inclusion criteria were excluded. For standardization and consistency, the reviewers independently reviewed the first 100 titles and abstracts and then met to review the level of agreement and discuss any differences that arose in the operationalization of the inclusion criteria. As the reviewers had a strong agreement in the inclusion/exclusion classification of the first 100 titles and abstracts, the rest of the titles and abstracts were divided between the two reviewers so each of the remaining records could be excluded based on only one screener's opinion. Both reviewers screened all the full texts of studies identified as potentially meeting the inclusion criteria based on the title and abstract. Full texts deemed to be included in the review had to be identified as fulfilling all inclusion criteria by both reviewers. The screening results were compared and any discrepancies were discussed until a consensus was reached. If consensus could not be reached, a third reviewer (R. P. E.) resolved those conflicts.

Titles and abstracts identified through citation chaining and experts were searched for in Covidence and any documents that had already been screened in Covidence were removed. The remaining titles, abstracts and full texts of studies were screened by the same reviewers (A. H. F., E. C. R., R. P. E.) using the same method as described above.

### 2.3 Data extraction

Following the identification of the final set of included studies, one reviewer extracted the data (A. H. F.) including: authors, year, country, timing of assessment, exposure and outcome variables, analyses including control variables and key findings. The data extracted were verified by the second reviewer (E. C. R.).

### 2.4 Statistical analyses

**Figure 1:**
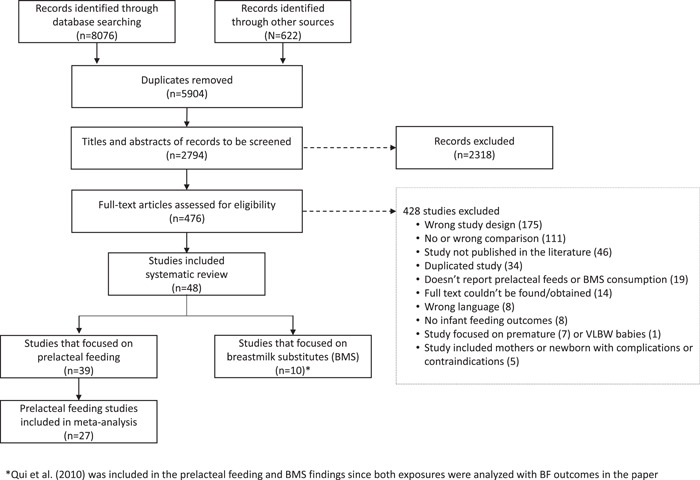
PRISMA diagram.

The results of the search and article selection are presented using the PRISMA flow diagram (Figure 1). Forty‐eight studies met the inclusion criteria for the systematic review

To assess the inclusion of the 48 studies in the meta‐analyses, they were all reviewed by two authors (P. A. R. N., J. S. V.). Studies were included in the meta‐analyses if they reported adjusted effect measures to assess the impact of the introduction of prelacteal feeds on BF outcomes of interest. Following a thorough review, 27 of the 48^1^ studies met the requirements for inclusion in the prelacteals meta‐analyses. However, 12 prelacteal feeding studies were not included in the meta‐analysis due to a lack of needed information on statistical parameters. Meta‐analyses were conducted on prelacteals regardless of type (milk‐ and water‐ based combined) and then milk‐based prelacteals only. Water‐based prelacteals by themselves could not be analysed as only one study was available (Qiu et al., 2007). It was not possible to conduct meta‐analyses for neonatal BMS introduction (between 4 days and 1 month postpartum) because in most studies the precise time of the introduction of BMS could not be ascertained.

As the length of follow‐up and BF practices assessed varied greatly across studies, outcomes were grouped into five categories: (1) any BF cessation among infants under 6 months of age; (2) EBF cessation among infants under 6 months of age; (3) any BF among infants under 6 months of age; (4) EBF among infants under 6 months of age; and (5) any BF cessation among infants up to 1 year of age.

A meta‐analysis was then conducted to test the effect of prelacteal feeds on each of these outcomes (Tables 1, S2 and S3, Supporting Information Appendix A). As such, a total of five separate meta‐analyses focused on prelacteal feeds and BF outcomes. If a study did not present BF outcomes that fit within these BF outcome categories, the study was not included in a meta‐analysis and was only reviewed in the narrative as part of the systematic review (Table S3).

**Table 1:** Summary of articles used for each meta‐analysis conducted on associations between prelacteals and BF outcomes.

Author	Year	Country	Outcomes				
			EBF cessation (<6 months)	EBF cessation (<6 months)	Any BF (<6 months)	Any BF (<6 months)	Any BF (<1 year)
			All forms	Milk‐based only	All forms	Milk‐based only	All forms
Agboado et al.	2010	England			X	X	
Alikasifoglu et al.	2001	Turkey	X				
Balogun et al.	2016	Nigeria	X				
Bruun et al.	2016	Denmark			X	X	
Chantry et al.	2014	United States	X		X	X	
Dashti et al.	2014	Kuwait			X		
Hruschka et al.	2003	Guatemala			X		
McCoy and Heggie	2020	United States			X	X	
McDonald et al.	2010	Australia	X	X	X	X	X
Parry et al.	2013	Hong Kong (China)					X
Patil et al.	2015	Bangladesh, Brazil, India, Nepal, South Africa, Tanzania, Pakistan, Peru	X				
Qiu et al.	2010	China			X		
Raghavan et al.	2014	India	X	X			
Raheem et al.	2014	Maldives			X	X	
Richard et al.	2021	Bangladesh, Brazil, India, Nepal, South Africa, Tanzania, Pakistan, Peru	X	X			
Semenic et al.	2008	Canada	X	X			
Sheehan et al.	1999	Canada			X	X	
Sheehan et al.	2006	Canada			X	X	
Tarrant et al.	2015	Hong Kong	X	X			X
Zarshenas et al.	2020	Iran			X	X	

Abbreviations: BF, breastfeeding; EBF, exclusive breastfeeding.

The included studies were carried out in different world regions, and evaluated different BF outcomes among populations of children of different ages. Hence, we pooled the estimates using both random effects and fixed effects models using Stata 17.0. The findings were similar for random and fixed effects models, hence only the results from random effects models are presented here. The *I* ^2^ was used to investigate heterogeneity and the funnel plot and Egger test were used to assess the occurrence of publication bias.

Some studies reported on different effect measures (either hazard ratio or odds ratio). Thus, sensitivity analyses were performed by limiting the models to studies that reported on the same impact measure (Supporting Information Appendix B).

### 2.5 Study quality assessment

**Figure 2:**
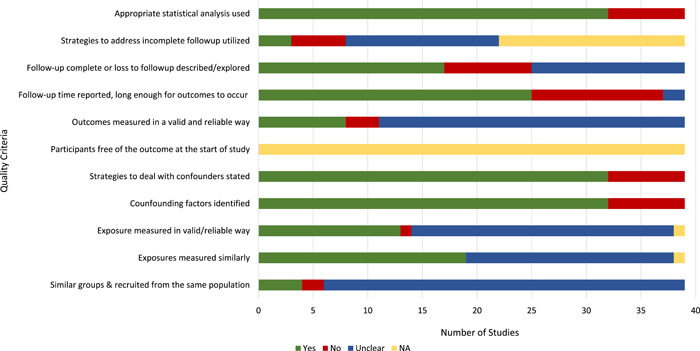
Assessment of study quality with Johan Briggs Institute protocol. Prelacteal feeds studies.

**Figure 3:**
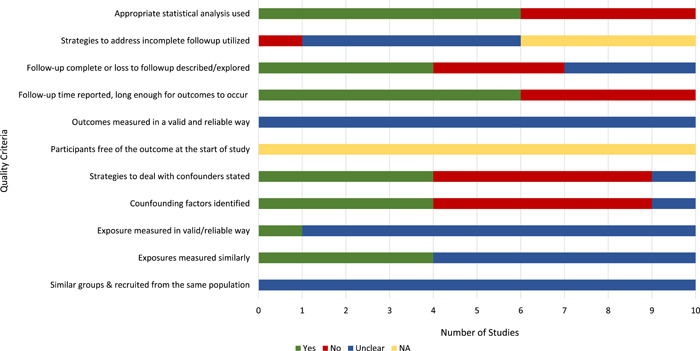
Assessment of study quality with Johan Briggs Institute protocol. Neonatal breast milk substitutes studies.

The Joanna Briggs Institute (JBI) critical appraisal tools for observational, quasi‐experimental and experimental studies was used to assess the quality of each study included in the review (Moola et al., 2015) (Figures 2 and 3 and Tables S4 and S5). For all study quality assessments, two authors (A. H. F., E. C. R.) were standardized against each other.

## 3 RESULTS

Of the 48 studies, 18 were conducted in North America, followed by 7 in East Asia and Pacific, 6 in Europe and Central Asia, 5 in the Middle East and North Africa, 5 in Latin America and the Caribbean, 3 in South Asia and 2 in Sub‐Saharan Africa. Thirty‐two studies were conducted in high‐income countries, 7 were conducted in upper‐middle‐income countries and 7 in LMICs. Finally, 2 studies were conducted in multiple lower‐ and upper‐middle‐income countries that spanned 3 regions, including South Asia, Latin America and the Caribbean, Sub‐Saharan Africa.

Out of the 48 studies, 40 were prospective cohort studies, 7 were RCTs and 1 was a longitudinal, quasi‐experimental study. Of the studies that focused on prelacteals, 20 focused on milk‐based prelacteals, 1 focused on water‐based prelacteals and 18 focused on prelacteals in any form (Agboado et al., 2010; Alikasifoglu et al., 2001; Balogun et al., 2016; Bruun et al., 2016; Cardoso et al., 2010; Chantry et al., 2014; Dashti et al., 2014; Demirci & Bogen, 2017; Dennis et al., 2014, 2019; Feinstein et al., 1986; Forster et al., 2006; Gray‐Donald et al., 1985; Hayek et al., 2019; Hossain et al., 1992, 1994; Hruschka et al., 2003; Lakati et al., 2010; McCoy & Heggie, 2020; McDonald et al., 2010; McKinney et al., 2016; Parry et al., 2013; Patil et al., 2015; Qiu et al., 2007, 2010; Raghavan et al., 2014; Raheem et al., 2014; Rasheed et al., 2009; Richard et al., 2021; Semenic et al., 2008; Sheehan et al., 2006, 1999; Tarrant et al., 2015; Vehling et al., 2018; Weisband et al., 2017; Zakarija‐Grkovic et al., 2016; Zarshenas et al., 2020) (Table 2). Of the studies that focused on the neonatal introduction of BMS, 7 focused on milk‐based BMS, 1 examined water‐based fluids and 2 examined BMS and/or water‐based fluids combined (i.e., any form) (Table 3). One study examined both prelacteals and neonatal BMS introduction and was included in both Tables 2 and 3 (Qiu et al., 2010). With regard to BF outcomes, 2 studies evaluated BF initiation, 17 evaluated EBF and 38 evaluated any BF. Four studies focused on other BF outcomes.

**Table 2:** Included studies that prospectively examined the association between prelacteals and BF outcomes.

Author (Year) Country	Population/methods	Type of exposure	BF outcomes	Results*
**Prelacteals**				
Agboado et al. (2010) England	2107 mothers participated in a BF peer support programme. Assessments were at 6 weeks, 17 weeks, 6 months and 9 months.	Milk‐based	BF duration BF cessation	Median duration of BF was shorter for infants receiving formula in hospital compared to those that didn't receive it (17 vs. 27 weeks). Higher risk of stopping breastfeeding was seen with mothers giving formula in the hospital compared with those who did not not (HR = 1.56; 95% CI, 1.36–1.78) (AdjHR = 1.50; 95% CI, 1.26–1.79).
Alikasifoglu et al. (2001) Turkey	91 mothers with healthy births participated in a brief BF support programme administered by nurses/doctors. Assessments were at the first well‐child visit and monthly for 4 months.	Any form	BF initiation EBF (4 months) EBF duration	BF was initiated later among infants given formula in hospital compared to their counterparts (4.4 ± 2.9 vs. 2.9 ± 1.4 h, respectively; *p* = .006). EBF among those supplemented vs had not supplemented in hospital (32% vs. 66%, respectively). EBF was longer among those that did not supplement vs those that did (χ^*2*^ = 10.35, *p* = 0.001). EBF duration negatively associated with receiving supplementary formula in the hospital (*β* = 0.3544; SE = 0.1427; Exponential *β* = 1.4253; 95% CI = 1.0775–1.8854).
Balogun et al. (2016) Nigeria	210 mothers were recruited during pregnancy and were assessed prenatally and at 6 weeks and 3 months postpartum.	Any form	EBF (3 months) EBF cessation (3 months)	Mothers who did not give pre‐lacteals at birth practiced EBF more than mothers who did give prelacteals (log‐rank test χ^2^ = 5.6; *p* = 0.02). Higher risk for discontinuing EBF was seen with mothers who fed prelacteals (HR = 2.12; 95% CI: 1.13–3.97) (AdjHR = 2.93; 95% CI: 1.49–5.77).
Bruun et al. (2016) Denmark	499 mothers were assessed via messaging at 3 days after birth and then continued weekly for 34 weeks.	Milk‐based	Early BF cessation (1–16 weeks)	Infants receiving formula supplementation in the first few days after birth were more likely to end BF early compared to those not supplemented (AdjOR = 3.13; 95% CI: 1.85–5.31).
Cardoso et al. (2010) Chile	201 mothers were assessed perinatally and 1 month postpartum.	Any form	EBF (1 month)	Risk of not EBF is associated with in‐hospital supplementation (RR = 1.55; 95% CI: 1.03‐2.34; Adj RR = 1.54; 95% CI: 1.01–2.35).
Chantry et al. (2014) United States	393 mothers completed the study and were assessed prenatally, within 24 h after birth, at Days 3, 7, 14, 30 and 60 postpartum.	Milk‐based	Not fully BF (30–60 days) BF cessation (60 days)	In‐hospital formula supplementation led to increased likelihood not be fully breastfeeding than in hospital EBF (67.8% vs. 36.7%; OR = 3.6; 95% CI: 2.4–5.5) (AdjOR = 3.9; 95% CI: 2.2–6.5) (AdjRR = 1.79; 95% CI: 1.43–2.27). In‐hospital formula supplementation led to increased likelihood of BF cessation by day 60 than in‐hospital EBF (32.8% vs. 10.5%; OR = 4.2; 95% CI: 2.4–7.1) (AdjOR = 4.4; 95% CI: 2.2–8.7) (AdjRR = 2.71; 95% CI: 1.75–4.53).
Dashti et al. (2014) Kuwait	345 mothers in a study of infant feeding practices were assessed in the hospital before discharge and at 6, 12, 18 and 26 weeks postpartum.	Any form	Full BF cessation Any BF cessation	Lower risk for discontinuing full BF for infants who did not receive prelacteal feeds during 6 months postpartum (HR = 0.69; 95% CI: 0.50–0.97). In adjusted analyses, there was no significant risk. Lower risk of discontinuing any BF for infants who did not receive prelacteal feeds during 6 months postpartum (HR = 0.42; 95% CI: 0.22–0.80). In adjusted analyses, there was no significant risk.
Demirci and Bogen (2017) United States	48 mothers in a study using a commercial infant‐feeding app were assessed at 2 and 8 weeks postpartum.	Milk‐based	EBF (2 weeks)	Infants receiving in‐hospital formula were less likely to be EBF than infants who received only human milk (OR = 0.3; 95% CI: 0.1–0.9).
Dennis et al. (2019) Canada	565 immigrant and Canadian‐born mothers were assessed within 1, 3, 6 and 12 months postpartum.	Any form	EBF (1, 3, 6 months)	In hospital formula supplementation led to decreased likelihood to be EBF at 1 month (AdjOR = 0.44; 95% CI: 0.28–0.68) and 3 months (AdjOR 0.45; 95% CI: 0.28–0.74). In hospital formula supplementation was not significantly associated with EBF across models (Model 1: OR = 0.90; 95% CI: 0.54–1.49); Model 2: OR = 1.07; 95% CI: 0.63–1.83). When infants received supplementation in the hospital, the log odds of EBF decreased at 6 months (*b*: −1.12; SE 0.24; *p* < 0.0001). In hospital supplementation associated with log odds of EBF was time‐dependent (*b* = 0.47, SE 0.16, P 0.003).
Feinstein et al. (1986) United States	196 mothers, 78% were black, were assessed monthly for the first 4 months postpartum.	Milk‐based	Any BF (4, 10, 16 weeks)	At all time periods, mothers who supplemented in the hospital (<1/day) were significantly more likely to continue BF than those who supplemented 1–4 or >4 times/day (4 weeks: 96%, 86%, 68%, respectively; 10 weeks: 96%, 71%, 44%, respectively; 16 weeks: 74%, 58%, 20%, respectively.
Forster et al. (2006) Australia	764 mothers who were included in the final analytical model, had been assessed prenatally, after birth and 6 months postpartum.	Milk‐based	Any BF (6 months)	Any BF at 6 months: Infants who received formula in the hospital were less likely to be fed any breast milk at 6 months. (OR = 0.39; 95% CI: 0.29–0.53) (AdjOR = 0.43; 95% CI: 0.3–0.62).
Gray‐Donald et al. (1985) Canada	621 mothers were included in a controlled clinical trial assessing effects of BF support to limit supplementation. Medical records were collected and mothers were interviewed at 9 weeks.	Any form	Any BF (4, 9 weeks)	Breastfeeding at 4 weeks and 9 weeks was associated with a higher likelihood of not having received in hospital formula supplementation (4 weeks: 19.4% vs. 4.4%, respectively, *χ* ^2^ = 14.49, *p* < 0.001) (9 weeks: 20.9% vs. 7.9%, respectively; *χ* ^2^ = 13.03, *p* < 0.001).
Hayek et al. (2019) Israel	2119 mothers were recruited and 1497 reported info on EBF. Mothers were assessed at birth, 2, 6, 12 and 24 months postpartum.	Any form	EBF duration	Infants being EBF were less likely to have received in‐hospital formula compared to those not EBF (52.2% vs. 71.8%, respectively). A 10% decrease in the duration of EBF was seen among infants given formula or pacifiers in hospital (Fully imputed time ratio = 0.92; 95% CI: 0.86–0.99).
Hossain et al. (1992) Egypt	152 infants were enroled. Mothers were assessed 3–4 days after birth, then twice weekly until 47 weeks.	Any form	BF initiation Overall BF, EBF duration	Newborns fed prelacteals had BF initiated later than those not fed prelacteals (mean 14 h vs. 2.1 h, respectively). Regardless of infants' prelacteal feeding status, age‐specific prevalence of overall BF declined similarly (100% at 0–3 weeks to 84% at 44–47 weeks). EBF rates were significantly higher among breastfed infants at 0–3, 4–7 and 8–11 weeks of age for those who did not receive prelacteal feeds compared to those that did.
Hossain et al. (1994) Egypt	152 mother/infant pairs were enroled. Mothers were assessed 3–4 days after birth, then twice weekly until 47 weeks.	Any form	EBF (<11 weeks)	6% of 0–11 months old infants who were fed prelacteals were EBF vs. 39% not fed prelacteals. Infants 0–11 weeks old who were fed prelacteals were less likely to EBF compared to those not fed prelacteals (AdjOR = 0.12; 95% CI: 0.04–0.37).
Hruschka et al. (2003) Guatemala	501 infants were enroled with 328 included in analyses. Mothers were assessed prenatally and every 2 weeks until 6 months postpartum.	Any form	Full BF cessation (6 months)	After adjusting for confounders, mothers who supplemented before the onset of lactation were at higher risk of ending full BF compared to those that did not feed supplements. (HR = 1.29; 95% CI: 0.97–1.71) (AdjHR = 1.49; 95% CI: 1.05–2.11).
Lakati et al. (2010) Kenya	691 mother/infant pairs were recruited before discharge and assessed monthly for the first 6 months.	Any form	Full BF (6, 10, 14, 19, 23 weeks) Full BF cessation	Infants who didn't have prelacteals were more likely to be fully BF at most time points (it was not significant only at 23 weeks). Infants who had prelacteal feeds had significantly higher odds for early complementary feeding (and hence early cessation of full BF) compared to counterparts (6 weeks: AdjOR = 56.3; Exp(B): 4.031 (10 weeks: AdjOR = 6.57; Exp(B): 1.873 (14 weeks: AdjOR = 6.47; Exp(B): 1.868 (19 weeks: AdjOR 9.84; Exp(B): 2.287.
Martin‐Calama et al. (1997) Spain	180 newborns were randomly assigned to receive glucose or be EBF. Mothers were assessed in the hospital and at 5 months.	Water‐based	Any BF (16, 20 weeks) BF duration	A higher rate of infants in the nonglucose water group was breastfed at 16 weeks than in the glucose water group. Not significant at 20 weeks. A longer BF duration was seen in the nonglucose water group compared to the glucose water group.
McCoy and Heggie (2020) United States	A matched sample of ethnic/racially diverse WIC mothers was analysed (*n* = 5310). Data was obtained from WIC appointments.	Milk‐based	BF duration	Infants EBF in the hospital had a longer BF duration compared to those who received in‐hospital formula (HR = 6.1; 95% CI: 4.9–7.5). As age increased, BF duration increased among those infants who were EBF in the hospital (1 month: HR = 4.1; 95% CI 3.5–4.7) (1–6 months: HR = 8.2; 95% CI 5.6–12.1) (>6 months: HR = 14.6; 95% CI 8.9–24.0).
McDonald et al. (2010) Australia	849 mothers were recruited and randomized to receive extended midwifery support. Mothers were assessed in hospital, at 2 and 6 months.	Milk‐based	Full BF cessation (<6 months) Any BF cessation (<6 months)	Infants introduced to in‐hospital artificial milk were more likely to stop full BF (AdjOR = 1.52; 95% CI: 1.09–2.12) or any BF (AdjOR = 1.64; 95% CI:1.14–2.35) before 6 months.
McKinney et al. (2016) United States	1636 mothers from a community‐based project were included in the sample. Mothers were assessed in‐hospital, 1 and 6 months. Medical records were used for infant feeding data.	Milk‐based	BF duration	In‐hospital formula introduction was the largest predictor of breastfeeding duration, even in models controlling for variables inkling race/ethnicity (*β* = −9.79; 95% CI: − 11.43 to −8.16).
Parry et al. (2013) Hong Kong (China)	1246 mother/infant pairs were included in the analysis. Medical records were abstracted for in‐hospital data, 1, 2, 3, 6, 9 and 12 months.	Any form	BF duration BF cessation	Infants who were EBF in the first 24 h of life BF for longer than those receiving formula (*p* < 0.001). Infants receiving formula in the first 48 h had a higher risk of stopping BF than those who did not (HR = 1.67; 95% CI: 1.42–1.98) (AdjHR = 1.51, 95% CI: 1.27–1.80)
Patil et al. (2015) (Bangladesh, Brazil, India, Nepal, South Africa, Tanzania, Pakistan, Peru)	2142 infants ≤17 days were enroled, assessed and followed up 2× each week through 24 months of age. Additional data on infant feeding was collected on a monthly basis.	Any form	Partial/no BF (1 month)	Infants given prelacteal feeds were more likely to be partially breastfeeding compared to those not given prelacteal feeds (AdjOR 1.48; 95% CI: 1.04–2.1)
Qiu et al. (2010) China	1520 mothers were enroled and assessed before hospital discharge and at 1, 3 and 6 months postpartum.	Any form	Any BF (6 months) EBF (6 months) Intro to infant formula (<3 months)	Breast milk as first food increased the likelihood of any BF at 6 months (77% vs. 71%). Breast milk as first food increases the likelihood of EBF at 6 months (51.4% vs. 40.4%). Infants whose first feed was not breast milk were 2.18 times more likely to be subsequently fed with formula compared to those infants whose first feed was breast milk (95% CI: 1.429–3.317).
Qiu et al. (2007) China	638 mothers were recruited and assessed before discharge and at regular intervals until their infants were 6 months of age.	Any form	Any BF on hospital discharge	Infants given in‐hospital prelacteals were less likely to be BF at discharge (AdjOR = 0.115; 95% CI: 0.055–0.238).
Raghavan et al. (2014) India	400 mother/infant dyads were enroled and assessed within 48 h of delivery and at 6 weeks. Medical record data were also obtained.	Milk‐based	EBF cessation (6 weeks)	EBF cessation at 6 weeks: infants given prelacteals (breast milk substitutes on Day 1) were at increased risk of stopping EBF at 6 weeks than their counterparts (RR = 2.72; 95% CI: 1.37–3.77) (AdjOR = 2.96; 95% CI:1.09–8.06).
Raheem et al. (2014) Maldives	458 mothers were recruited prenatally and assessed at 36 weeks during pregnancy and 1, 3 and 6 months postpartum.	Milk‐based	BF cessation (<6 months)	Infants given formula were more likely to stop BF before 6 months compared to those not given formula (AdjOR = 6.0; 95% CI: 1.64–21.8).
Rasheed et al. (2009) Bangladesh	1472 mother/infant dyads were included in the analysis. Data were collected from mothers monthly on infant food consumption.	Any form	Full BF trajectory (6 months) Continuous mixed feeding trajectory (4 months)	Infants offered prelacteals were more likely to be FBT compared to the intermittent feeding trajectory (IFT) (Fully adjusted model: AdjOR = 1.76; 95% CI: 1.06–2.9) Infants prelacteals were not more likely to be in the CMFT compared with IFT (Fully adjusted model: AdjOR = 0.92; 95% CI: 0.70–1.20).
Richard et al. (2021) Bangladesh, Brazil, India, Nepal, South Africa, Tanzania, Pakistan, Peru	Data for these analyses included 1470 infants and was limited to the first month of age and obtained at enrolment, surveillance visits and Month 1 visit. The full study was extended until the child was 24 months old.	Any form	Transitioning to partial BF (<6 months)	Prelacteal feeding was not associated with the risk of transitioning to partial BF before 6 months (HR = 1.14; 95% CI: 0.94–1.4) (AdjHR = 1.18; 95% CI: 0.96–1.44).
Samuels et al. (1985) United States	632 mothers were enroled, 417 chose to BF and were included in the analyses. Data consisted of hospital medical records plus paediatric records through 4 months.	Milk‐based	BF duration (4 months) BF cessation (4 months)	Formula received in the hospital negatively influenced BF duration up to 4 months (*β* = −0.395). A lower proportion of breastfed infants who were receiving formula in the hospital were BF at 4 months compared to those breastfed infants who did not receive formula in the hospital (40% vs. 70%, respectively) (*n* = 417).
Semenic et al. (2008) Canada	189 mothers began the study and were assessed between 24–72 h after birth, then at 6 weeks, 4 months, 6 months postpartum.	Milk‐based	EBF (6 months)	In‐hospital formula supplementation shortened the duration of EBF to 6 months (Unadjusted: *β* = 0.48; Adj: *β* = 0.34) (AdjHR = 1.4; 95% CI: 1.01–1.96).
Sheehan et al. (1999) Australia	179 mothers were recruited, with 154 completing the trial. Mothers were assessed in the hospital postpartum and then every 4 weeks up to 25 weeks.	Milk‐based	EBF (hospital discharge) BF duration	In‐hospital supplementation did not make a difference in EBF after hospital discharge (*U* scores = 2065.5, *Z* scores = −1.17, *p* = 0.24). In‐hospital supplementation did not make a difference in BF duration after hospital discharge. (*U* scores=2036.5, *Z* scores = −1.49, *p* = 0.14).
Sheehan et al. (1999) Canada	227 mothers completed the first survey. Mothers were assessed in‐hospital and 6–8 weeks. Medical records were also extracted.	Milk‐based	BF (≥6 weeks)	Infants not receiving in‐hospital supplementation were more likely to BF ≥ 6 weeks, compared to those who supplemented (79.6% vs. 61.1%, respectively, *p* = 0.005) (OR = 2.49; 95% CI: 1.25–4.98).
Sheehan et al. (2006) Canada	1250 mothers were recruited and 890 completed the study. Mothers were assessed at hospital discharge and 4 weeks.	Milk‐based	BF cessation (by 4 weeks)	Infants receiving in‐hospital supplementation stopped BF by 4 weeks compared to not supplemented (22% vs. 8.8%, respectively; *p* < .001) (OR = 2.94; 95% CI: 1.97–4.50) (AdjOR = 2.4; 95% CI: 1.39–4.17).
Tarrant et al. (2015) Hong Kong	2560 mothers‐infant pairs were included in the final analyses. Mothers were assessed in‐hospital follow‐up was at 1, 2, 3, 6, 9 and 12 months.	Milk‐based	Any BF cessation EBF cessation	Infants who were high, medium, or low partially BF in‐hospital had an increased risk of BF cessation compared to EBF. (High‐partially: AdjHR = 1.29; 95% CI: 1.14–1.46) (Medium‐partially: AdjHR = 1.68; 95% CI: 1.49–1.90) (Low‐partially: AdjHR = 1.73; 95% CI: 1.39–2.16). Infants who were high‐, medium‐ and low‐partially BF had an increased risk of EBF cessation compared to those EBF. (High‐partially: AdjHR = 1.22; 95% CI: 1.10–1.36) (Medium‐partially: AdjHR = 1.47; 95% CI: 1.32–1.64) (Low‐partially: AdjHR = 1.69; 95% CI: 1.38–2.07).
Vehling et al. (2018) Canada	2285 mother/infant dyads were included in the final analysis. Mothers were assessed in hospital and at 3, 6, 12, 18 and 24 months.	Milk‐based	BF duration BF cessation	Infants who were EBF in hospital had a longer median duration of any BF than infants who received in‐hospital formula (11 vs. 7 months, respectively, *p* < 0.001). In‐hospital EBF was associated with a reduced risk of BF cessation over time (HR = 0.73; 95% CI: 0.66–0.81) (AdjHR = 0.79; 95% CI: 0.71–0.87).
Weisband et al. (2017) United States	The study recruited mothers with GDM and those without, but only mothers without GDM (*n* = 2139) were included in this review. Mothers were assessed prenatally and 10 times over a 1‐year period.	Any form	Any BF duration	Among women without GDM, no in‐hospital supplementation was associated with longer breastfeeding duration (Unadjusted *β* = 10.4 weeks; 95% CI: 8.6–12.2) (Adj *β* = 10.1 weeks; 95% CI: 8.3–11.8; *p* < 0.001).
Zakarija‐Grkovic et al. (2016) Croatia	773 mothers were included in the study and assessed at birth and 3, 6, 12 and 24 months (nurses also recorded hospital feeding data during hospitalization).	Milk‐based	EBF (3 and 6 months) Any BF (3 and 6 months)	Infants who received in‐hospital supplementation were less likely to be EBF at 3 months compared to their counterparts (AdjOR = 0.567; 95% CI: 0.358–0.897). No significant association at 6 months (AdjOR = 0.489; 95% CI: 0.232–1.033). Infants who received in‐hospital supplementation were less likely to be BF at 3 months compared to their counterparts (AdjOR = 0.549; 95% CI: 0.326–0.924). No significant association at 6 months (AdjOR = 1.459; 95% CI: 0.808–2.634).
Zarshenas et al. (2020) Iran	700 mothers were recruited and assessed within 48 h after birth and at 4, 12, 16 and 26 weeks postpartum.	Milk‐based	Full BF cessation (<26 weeks) Any BF cessation (<26 weeks)	Infants who received in‐hospital supplementation were more likely to have stopped full BF before 26 weeks (HR = 3.54; 95% CI: 2.93–4.28) (AdjHR = 3.15; 95% CI 2.59–3.83). Infants who received in‐hospital supplementation were more likely to have stopped any BF before 26 weeks (HR = 1.91; 95% CI: 1.27–2.88) (AdjHR = 1.65; 95% CI: 1.08–2.52).

Abbreviations: BF, breastfeeding; CI, confidence interval; HR, hazard ratio; OR, odds ratio.

* Results are significant at the *p* < 0.05 level unless otherwise indicated.

**Table 3:** Included studies that prospectively examined the association between breast milk substitutes and BF outcomes.

Author (Year) Country	Population/methods	Type of exposure	BF outcomes	Results*
**Breast milk substitutes**				
Barría et al. (1990) Chile	365 infants were included in the life‐table analysis to determine the influence of supplementation on lactation.	Milk‐based	Exclusive lactation	Median natural exclusive lactation was 10 months among infants not supplemented versus 3 months among infants supplemented with formula by 1 month postpartum.
Bunik et al. (2010) United States	341 mothers agreed to participate in the RCT but analyses were conducted only with the intervention group (*N* = 107). Mothers were assessed at discharge, daily for the first 2 weeks, then 1, 3 and 6 months postpartum.	Milk‐based	BF (1, 3, 6 months)	Mothers reporting 0–2 formula feedings at day 4 (0–2) were more likely BF at 1 month (OR = 7.7; 95% CI: 2.4–24.3), 3 months (OR = 3.1; 95% CI: 1.0–9.8) and 6 months (OR = 8.1; 95% CI: 1.0–65.2) compared with mothers reporting three or more supplementing feedings.
Dennis et al. (2014) Canada	265 mothers were recruited from 12 hospitals and were assessed in hospital, then at 1 and 16 weeks. Medical record data was also obtained about hospitalization.	Any form	EBF (16 weeks)	EBF at 1 week postpartum was significantly related to EBF at 16 weeks for migrant women (59.6% EBF vs. 40.4% no EBF) and Canadian‐born women (75.8% EBF vs. 24.2% no EBF). Infants not EBF at 1 week post‐partum (AdjOR = 0.26, 95% CI: 0.18–0.38) were more likely to not be EBF among migrant women (AdjOR = 0.26; 95% CI: 0.13–0.50) and Canadian women (AdjOR = 0.26; 95% CI: 0.18–0.38).
Flaherman et al. (2013) United States	40 EBF infants participated in an RCT. Mothers were assessed at 1 week and 1, 2 and 3 months postpartum.	Milk‐based	EBF (3 months)	18% of infants who received formula at 1 week were EBF at 3 months compared to 81% of infants who did not receive formula at 1 week, 81% (*p* < 0.001).
Giovannini et al. (2005) Italy	2450 mothers participated in the first interview. Mothers were assessed ≤1, 3, 6, 9 and 12 months after birth.	Milk‐based	Predominant BF	Infants introduced to formula within the first month of delivery were more likely to be predominately BF rather than EBF (AdjOR = 1.54; 95% CI: 1.14–2.09).
Grossman et al. (1990) United States	97 mothers were enroled in the intervention or control group and were assessed in the hospital and at 6 weeks. Mothers still nursing at 6 weeks were contacted monthly until weaning occurred.	Any form	BF cessation (<6 weeks)	A higher percentage of infants who were weaned at <6 weeks had received supplements ≤2 weeks after birth compared to those who were BF at 6 weeks (63% vs. 38%). There was no difference in the percentage of infants weaned at <6 weeks and those who were BF at 6 weeks that had received supplements between 2–4 weeks after birth (34% vs. 33%).
Hill et al. (1997) United States	Two cohorts of mothers were assessed at the following intervals: Phase I (*n* = 102): 1, 2, 3, 4, 5, 6, 8, 12, 16 and 20 weeks. Phase II (*n* = 176): 1 week, every 2 weeks until 20 weeks.	Milk‐based	BF (4, 8, 12, 16, 20 weeks)	Across all weeks, a significantly higher proportion of mothers in both samples who EBF were still BF compared with those who supplemented with formula.
Marques et al. (2001) Brazil	289 mother‐infant pairs complete the full follow‐up where mothers were assessed in the hospital within 24 h of delivery after milk was established and 2× a week for 12 months.	Milk‐based (with or without cereal)	BF duration	Mothers who introduced other milk in the first month had a shorter median duration than those who did not (65 vs. 165 days, respectively, *p* < 0.001).
Pérez‐Escamilla et al. (1993) Mexico	165 mothers were assessed in the hospital after delivery and 8, 70 and 135 days postpartum.	Milk‐based	BF (2, 4 months)	Mothers who were fully BF at 1 week postpartum were more likely to BF at 2 months than those partially BF (AdjOR = 4.6, 95% CI: 1.3–15.8). Mothers fully BF at 1 week postpartum were more likely to BF at 4 months than those partially BF (AdjOR: 4.1, CI: 1.7–10.0).
Qiu et al. (2010) China	1520 mothers were enroled and assessed before hospital discharge and at 1, 3 and 6 months postpartum.	Water‐based	Any BF (6 months) Any BF cessation	Feeding water within 1 month decreases the likelihood of any BF at 6 months (65% vs. 80%, respectively, *p* = < 0.001). Infants given water before 1 month of age were at higher risk of stopping any BF than those who were given water after 1 month (AdjHR = 1.713; 95% CI: 1.290–2.274)

Abbreviations: BF, breastfeeding; CI, confidence interval; EBF, exclusive breastfeeding; HR, hazard ratio; OR, odds ratio; RCT, randomised controlled trial.

* Results are significant at the *p* < 0.05 level unless otherwise indicated.

### 3.1 Prelacteals and exclusive BF cessation

**Figure 4:**
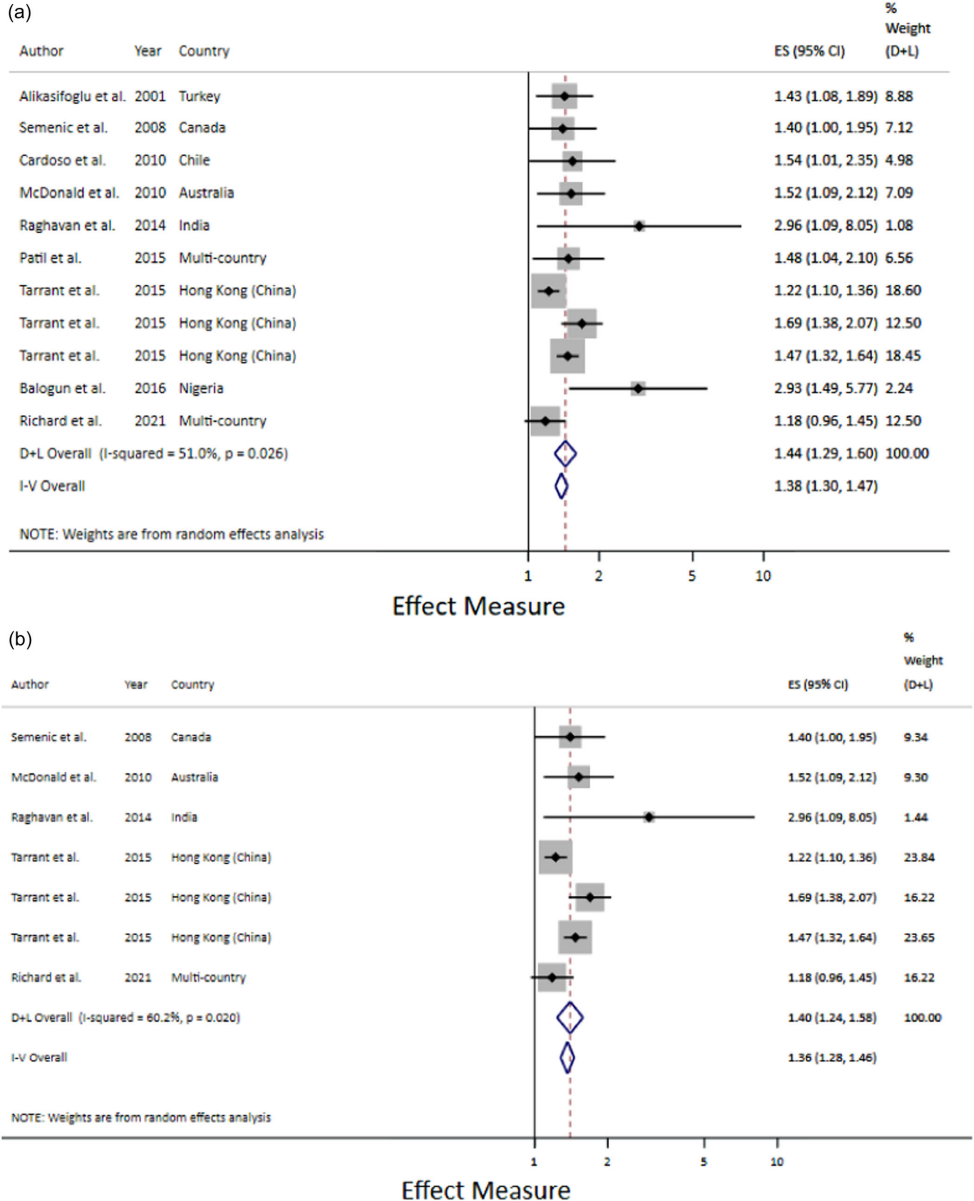
(a) Prelacteal feeds and exclusive breastfeeding cessation among infants under 6 months. (b) Prelacteal feeds and exclusive breastfeeding cessation among infants under 6 months. Only studies reporting impact measures for milk‐based prelacteals.

There was a strong relationship between prelacteal feeds and EBF cessation among infants under 6 months old when the prelacteals were analysed together regardless of type relative risk [RR] 1.44; 1.29–1.60) (Figure 4a) and the relationship remained when only milk‐based prelacteals were included (RR 1.40; 1.24–1.58) (Figure 4b).

### 3.2 Prelacteals and BF cessation

Prelacteal feeds were also a risk factor for any BF cessation by 6 months post‐partum when the prelacteals were analysed together regardless of type (Figure 5a) and the relationship remained when only milk‐based prelacteals were included (RR 2.23; 1.63–3.06) (Figure 5b). Prelacteal feeds were also a risk factor for any BF cessation by 1 year post‐partum (HR 2.02; 1.29–3.17) (Figure 5c).

Figure 5(a) Prelacteal feeds and any breastfeeding cessation among infants under 6 months. (b) Prelacteal feeds and any breastfeeding cessation among infants under 6 months. Only milk‐based prelacteals. (c) Prelacteal feeds and any breastfeeding cessation by 1 year.

**Figure:**
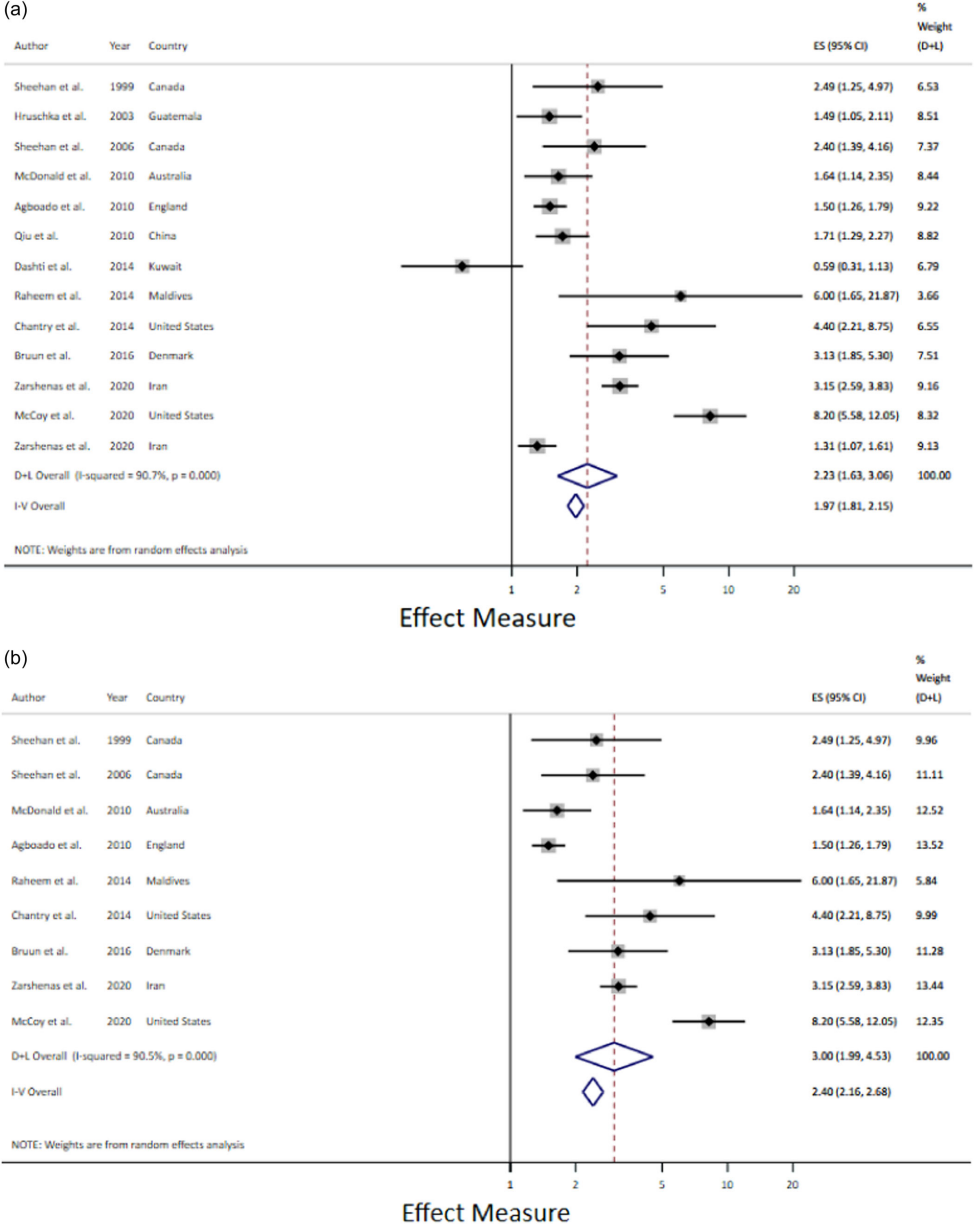

**Figure:**
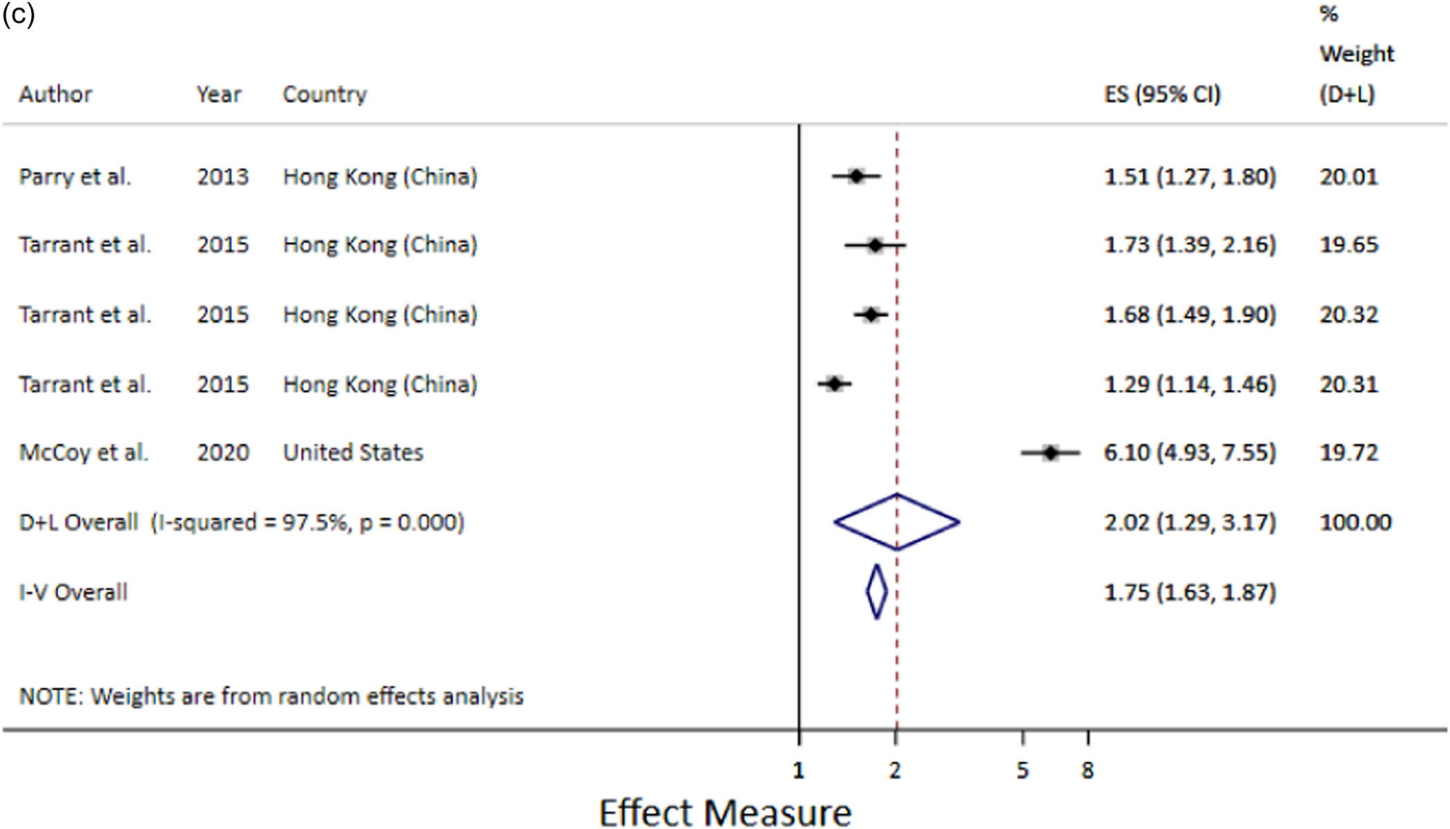

### 3.3 Sensitivity analysis

We were able to perform this analysis for any and EBF cessation under 6 months only, as for the other outcomes analysed the same impact measure was reported in the studies (either hazard ratio or odds ratio) (Supporting Information Appendix B). When limiting the analysis to articles that reported on the same effect measure, our findings remained: any BF cessation under 6 months, HR: 1.90, 95% CI: 1.23–2.95; EBF cessation under 6 months, HR: 1.41 95% CI: 1.24–1.61.

### 3.4 Prelacteal feeds and BF outcomes of studies not included in the meta‐analysis

As indicated above, 12 prelacteal feeding studies were not included in the meta‐analysis due to a lack of needed information on statistical parameters (Table S3). Ten of those studies found statistically significant inverse associations between prelacteal feeds and suboptimal BF outcomes. Of these, 4 were inversely associated with prevalence or duration of EBF (Demirci & Bogen, 2017; Hayek et al., 2019; Hossain et al., 1992; Vehling et al., 2018), 1 with full BF (Lakati et al., 2010) and 4 with any BF (Gray‐Donald et al., 1985; Samuels et al., 1985; Weisband et al., 2017). In 1 US study, the frequency of in‐hospital formal supplementation was inversely associated with BF duration (Feinstein et al., 1986). In another US study, prelacteal feeds were identified as the mediators between minority ethnicity/race (Black compared with White mothers) and short BF duration (McKinney et al., 2016). In the remaining 2 studies, an inverse relationship between prelacteals and BF outcomes was not found (Rasheed et al., 2009; Sheehan et al., 1999).

### 3.5 Early introduction of BMS and BF outcomes

Studies found that BMS introduction was associated with a lower likelihood of any BF at 1 (Bunik et al., 2010; Hill et al., 1997), 2 (Hill et al., 1997; Pérez‐Escamilla et al., 1993) months post‐partum, and a lower likelihood of EBF at 3 (Flaherman et al., 2013; Giovannini et al., 2005) and 4 (Dennis et al., 2014; Hill et al., 1997) months post‐partum. Likewise, there was a strong association between early BMS introduction and shorter any BF (Marques et al., 2001) and predominant BF (Barría et al., 1990) duration.

### 3.6 Risk of publication bias

The analysis of funnel plots suggests a small or no publication bias for most outcomes analysed, except for any BF cessation under 6 months and EBF under 6 months (Supporting Information Appendix C) that were indicative of a significant presence of bias. However, the Egger test was not statistically significant for any outcome investigated (any BF cessation under 6 months: *p* = 0.415; EBF cessation under 6 months: *p* = 0.071; any BF under 6 months: *p* = 0.896; EBF under 6 months: *p* = 0.845; any BF cessation up to 1 year: *p* = 0.296).

### 3.7 Quality assessment

The quality assessments showed that most studies (prelacteals and BMS) did not clearly describe whether the exposure or outcomes were measured in a valid or reliable manner. Most studies identified confounding factors and many of these used strategies to deal with them through the study design and/or data analysis. All studies used appropriate statistical analyses; however, several studies could have used more powerful analyses to draw conclusions (Figures 2 and 3).

## 4 DISCUSSION

Our findings indicate that milk‐based prelacteal feeds and early introduction of BMS during the neonatal period are risk factors for shorter EBF duration and any BF duration. Prelacteals are broadly classified as water‐based and milk‐based and this distinction is important for understanding the reasons for introducing them as well as the consequences that they may have on BF outcomes. While milk‐based prelacteals are often introduced as breast milk substitutes in response to self‐reported insufficient milk (McKenna & Shankar, 2009), water‐based prelacteals are typically given for biomedical (in‐hospital glucose water), perception of infant thirst and/or ritualistic reasons (intestinal cleansing with herbal concoctions, first sweet taste with honey drops) (Akuse & Obinya, 2002; McKenna & Shankar, 2009). For example, in India prelacteal feeds can include honey, jaggery (brown sugar from sugar cane), ghee (clarified butter) and ghutti (herbal paste). The choice of prelacteals may be specific to religion (e.g., Hinduism, Muslim), caste or family. Ritualistic prelacteals may be prepared with herbs such as cumin, cardamom, nutmeg, asafetida, caraway, cinnamon and aniseed. Ritualistic prelacteals may be given to a newborn by a person who has a special status within the family or community (McKenna & Shankar, 2009) and are usually given in small amounts and just on a few occasions. For these reasons, the design of BF support and education interventions should be based on strong formative research documenting cultural beliefs surrounding the perceived need for prelacteal feeds or BMS across the world.

Our findings also demonstrated overwhelming evidence that early introduction of BMS is a risk factor for shorter EBF and any BF duration. These findings confirm our hypothesis that the introduction of BMS during the neonatal period, oftentimes as a result of lactation problems related to lack of BF counselling and support during the establishment of the milk supply, is likely to disrupt nursing patterns, milk production and subsequent BF failure as a result (Pérez‐Escamilla et al., 2019; Vilar‐Compte et al., 2022).

Our study had several limitations. First, we could not answer all original questions listed in the protocol by type of prelacteal supplement because only one study included water‐based prelacteal supplements only (Qiu et al., 2007). Second, we could not conduct a meta‐analysis for neonatal BMS introduction as it was not possible to ascertain the precise time when these were introduced during the neonatal period, as specified in the systematic review protocol. Another potential limitation of the meta‐analyses is that 12 of the 39 articles focusing on prelacteal feeding could not be included because they did not report key statistical information. This may have been in part due to the fact that many of these articles did not examine the relationship between prelacteals and BMS as their primary aim.

There were also notable strengths. Our findings are based only on results from prospective studies, which are much less likely to be affected by recall bias compared with cross‐sectional and retrospective studies. The included articles represent different world regions, over one‐quarter of them were identified through citation chaining and we reviewed studies in three languages. Furthermore, we conducted sensitivity analyses and found no evidence of publication bias. The findings from the studies not included in the prelacteals meta‐analysis were consistent with the directionality of associations found in the meta‐analysis; that is, they also identified prelacteals as a risk factor for suboptimal BF outcomes. Indeed, 10 of the 12 articles excluded from the meta‐analysis found statistically significant inverse relationships between prelacteal feeds and diverse BF outcomes consistent with the results of the meta‐analysis.

Our systematic review and meta‐analysis have important policy implications as previous studies have identified modifiable risk factors, such as caesarean‐section delivery, unsupportive BF maternity practices and health care providers' lack of knowledge about how breast milk supply gets established, which can be addressed through health care facility improvements as well as BF counselling interventions (Kavle et al., 2017; Kavle, Ahoya, et al., 2019; Kavle, Picolo, et al., 2019; Pérez‐Escamilla et al., 2016, 2020; Rollins et al., 2016; Segura‐Pérez et al, 2022; World Health Organization & UNICEF, 2018; World Health Organization, 2018). Hence, our findings have strong implications for strengthening health care services prenatally, perinatally and during the neonatal period (Pérez‐Escamilla et al., 2019).

Moving forward, experimental interdisciplinary research studies are needed to identify the most promising interventions to address the risk factors for prelacteal feeding and the introduction of BMS during the neonatal period. To optimize the successful implementation of these interventions, it is important to codesign them with the target population and the providers serving them, following a health care systems implementation framework that takes the social determinants of health into account (Boccolini et al., 2015; Kavle et al., 2017; Nguyen et al., 2020; Pérez‐Escamilla & Sellen, 2015; Tomori et al., 2022). We found no prospective studies conducted in low‐income countries. There is an urgent need to conduct prospective studies to understand the effect of prelacteal feeds and neonatal introduction of BMS on BF outcomes in these settings, particularly given that BF is critical for promoting optimal maternal and child health outcomes in low‐income countries where women and children are at high risk of adverse outcomes for which BF is protective. More prospective studies are needed to improve our understanding of the association between water‐based prelacteals and BF outcomes. Lastly, studies need to clearly explain how they defined and measured prelacteal and later neonatal BMS feeding (Neves et al., 2022).

## 5 CONCLUSION

In conclusion, this review found that the introduction of BMS during the early and late neonatal periods is a statistically significant risk factor for shorter BF duration. Effective interventions are needed to prevent the introduction of unnecessary milk‐based prelacteals and BMS during the perinatal and neonatal periods to improve BF outcomes.

## AUTHOR CONTRIBUTIONS

Rafael Pérez‐Escamilla conceptualized and drafted the first version of the systematic review and meta‐analysis protocol and wrote the initial draft of the full manuscript. Amber Hromi‐Fiedler and Elizabeth C. Rhodes reviewed titles, abstracts and full texts of studies, created synthesis tables of studies included in the review and conducted the quality assessment of included studies. They also contributed to conceptualizing and drafting the systematic review and meta‐analysis protocol and manuscript. Rafael Pérez‐Escamilla provided guidance in reaching consensus on inclusion of studies as needed and extracted the data of studies published in Portuguese or Spanish. Paulo A. R. Neves contributed to conceptualizing and drafting the protocol for the systematic review and meta‐analysis and led the meta‐analysis in partnership with Juliana Vaz. They both critically reviewed the manuscript. Sofia Segura‐Pérez critically reviewed the protocol for the systematic review and meta‐analysis and the manuscript draft. Kate Nyhan developed and tested the search strategy, conducted the search and contributed to selecting a quality assessment tool. All authors read and approved the submitted manuscript.

## CONFLICTS OF INTEREST

The authors declare no conflicts of interest.

## Supporting information

Supporting information.

Supporting information.

Supporting information.

Supporting information.

Supporting information.

Supporting information.

Supporting information.

Supporting information.

## Data Availability

The authors will make the data of this study available upon reasonable request.

## Footnotes

1 This includes the one study that included both prelacteals and BMS. Only the prelacteal associations in this study were included in this meta‐analysis.
