# Supplementary material for: Impact of prelacteal feeds and neonatal introduction of breast milk substitutes on breastfeeding outcomes: A systematic review and meta‐analysis
Source: Matern Child Nutr. 2022 Apr 30;18(Suppl 3):e13368. doi: 10.1111/mcn.13368 (PMC9113480; doi:10.1111/mcn.13368)

**Online Appendix B. Sensitivity analysis of meta-analysis examining relationship between prelacteal feeds and breastfeeding outcomes.**

Figure 1S. Sensitivity analysis examining relationship between prelacteal feeds and any breastfeeding cessation under six months.


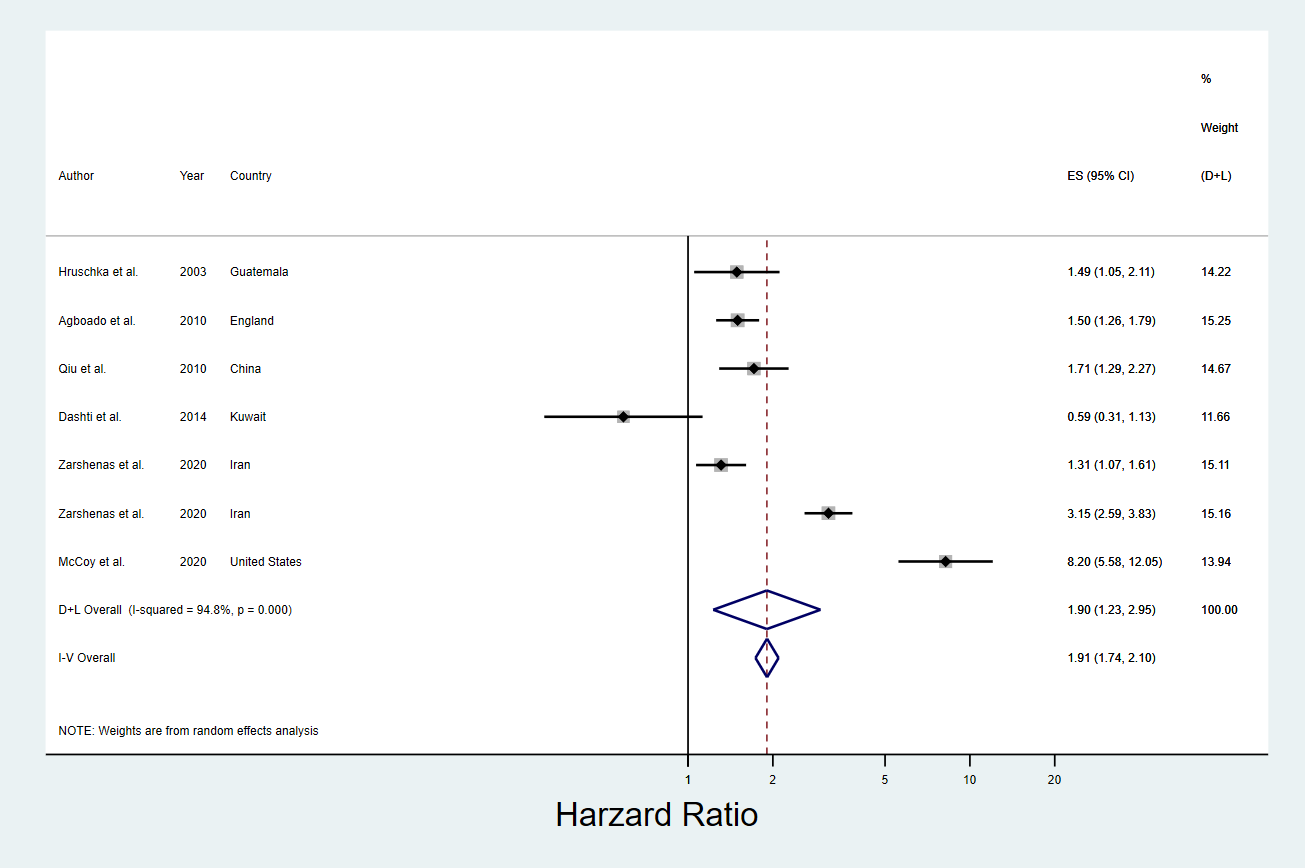


Figure 2S. Sensitivity analysis examining relationship between prelacteal feeds and any breastfeeding under six months.


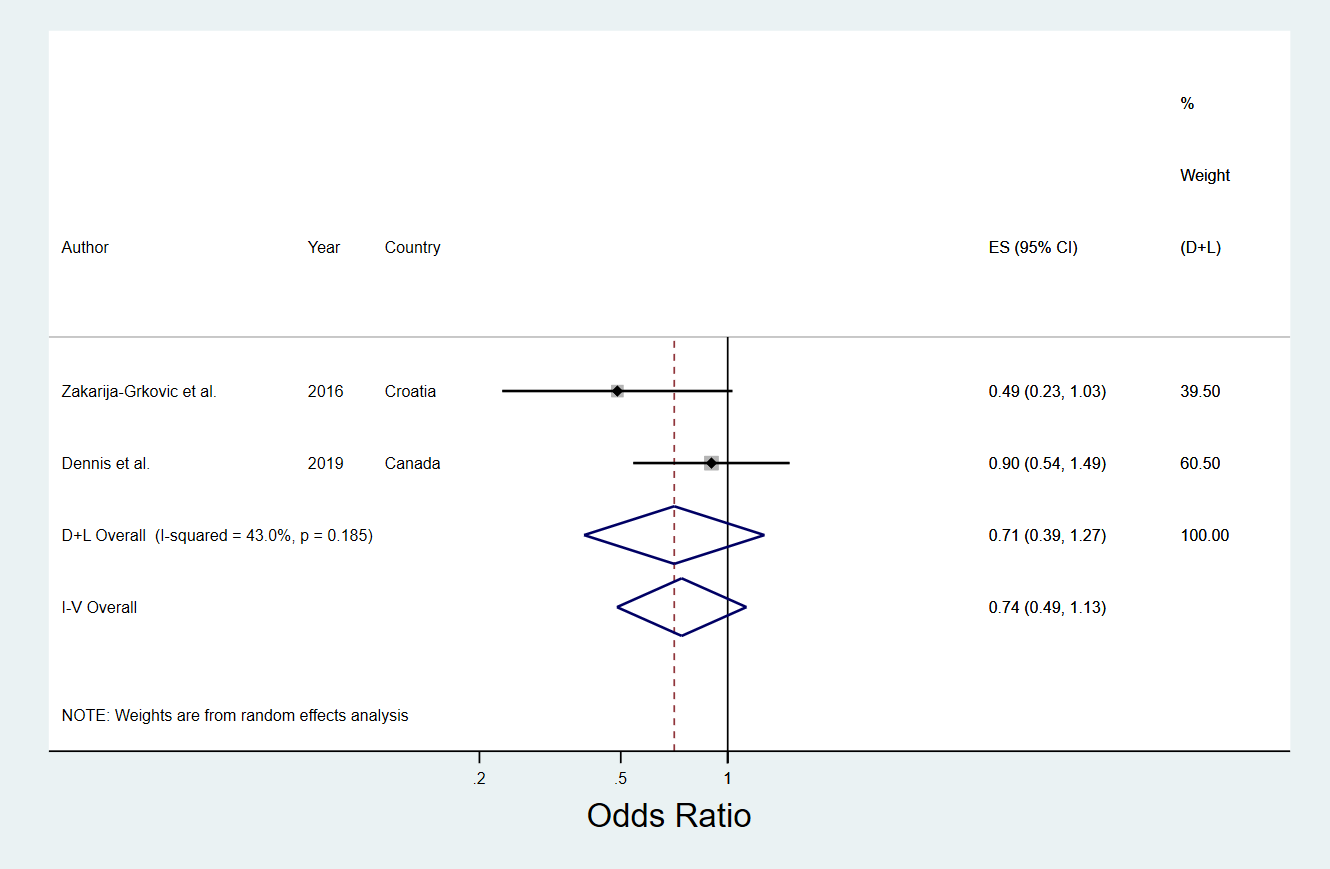


Figure 3S. Sensitivity analysis examining relationship between prelacteal feeds and exclusive breastfeeding cessation under six months.


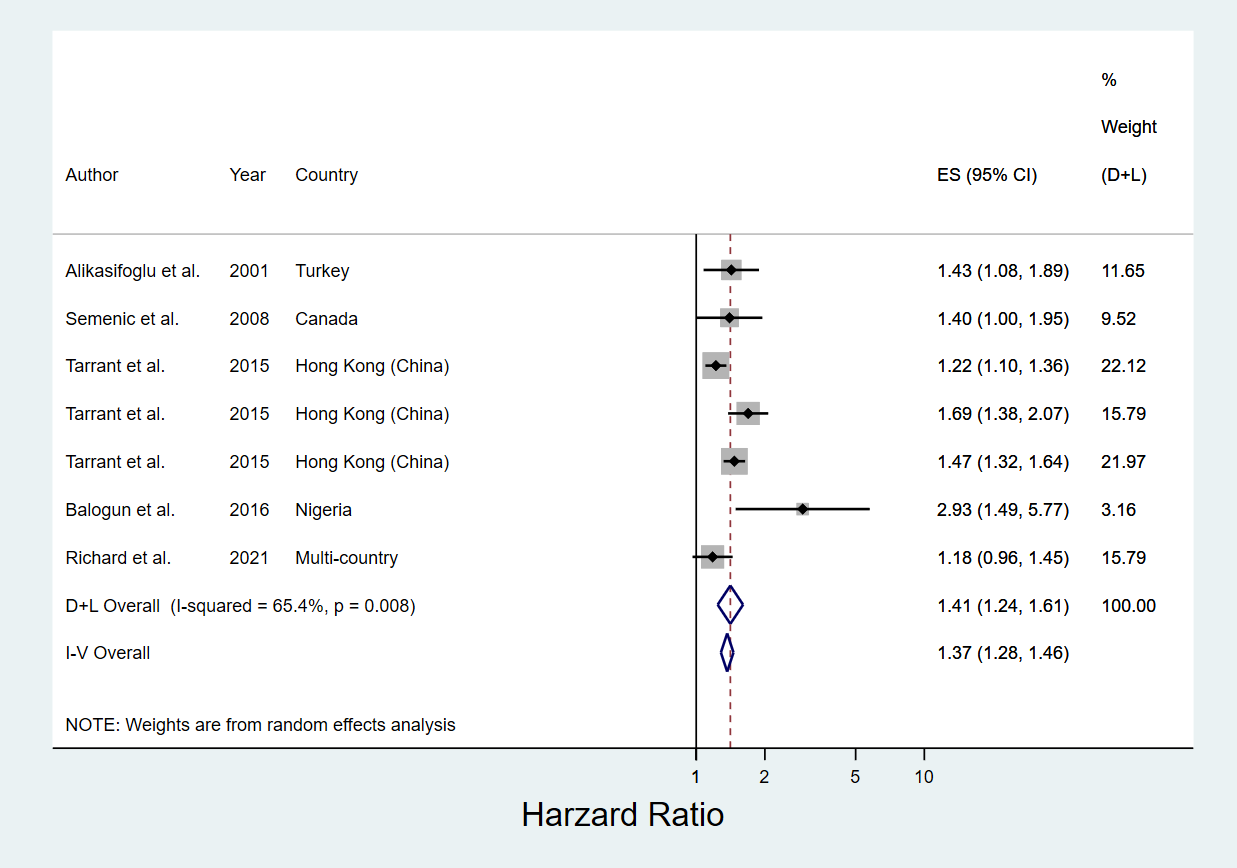


Figure 4S. Sensitivity analysis examining relationship between prelacteal feeds and exclusive breastfeeding cessation under six months.


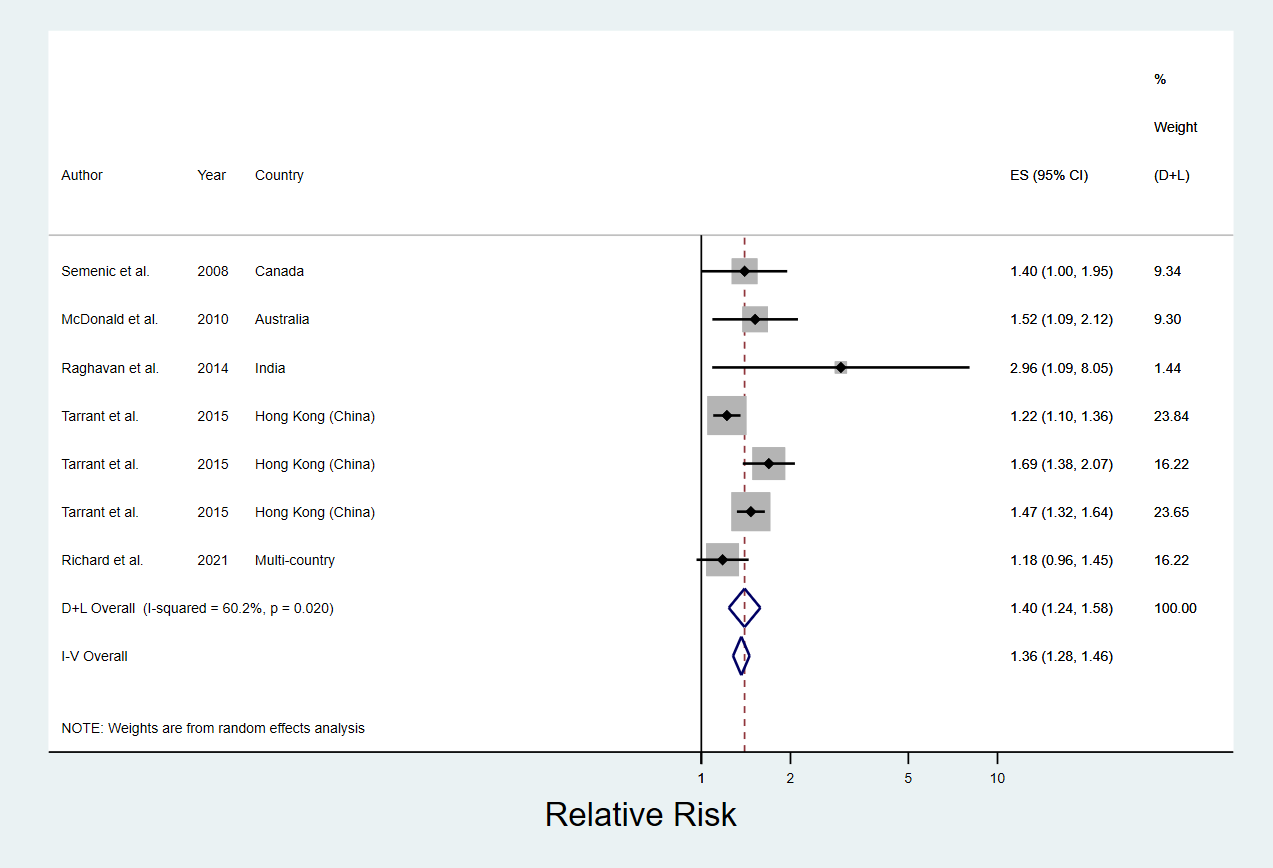

Supplement: Supplementary file 2 — Supporting information. [file MCN-18-e13368-s006.docx]
