# Supplementary material for: Impact of prelacteal feeds and neonatal introduction of breast milk substitutes on breastfeeding outcomes: A systematic review and meta‐analysis
Source: Matern Child Nutr. 2022 Apr 30;18(Suppl 3):e13368. doi: 10.1111/mcn.13368 (PMC9113480; doi:10.1111/mcn.13368)
Supplement: Supplementary file 3 — Supporting information. [file MCN-18-e13368-s003.docx]

**Online Supplementary Material C.** Funnel plots of meta-analysis examining relationship between prelacteal feeds and breastfeeding outcomes.

**Figure 1S. Funnel plot examining relationship between prelacteal feeds and any breastfeeding cessation under six months.**


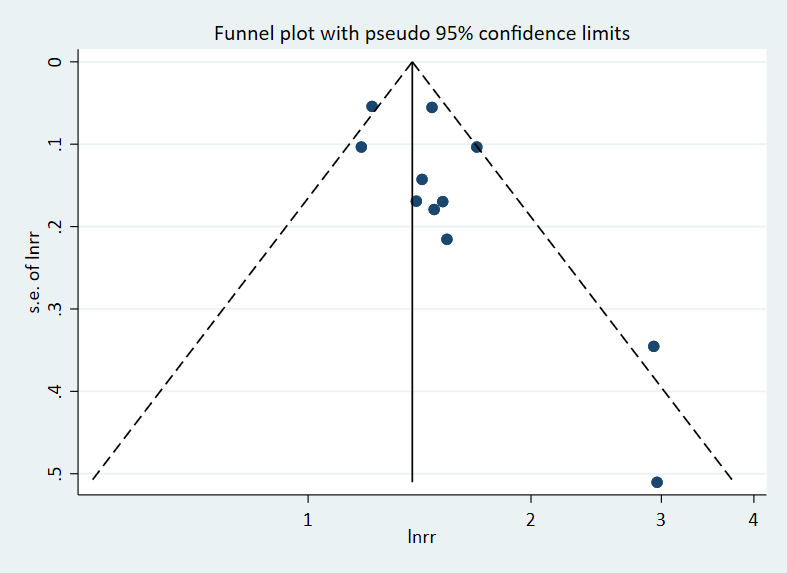


**Figure 2S. Funnel plot examining relationship between prelacteal feeds and exclusive breastfeeding cessation under six months.**


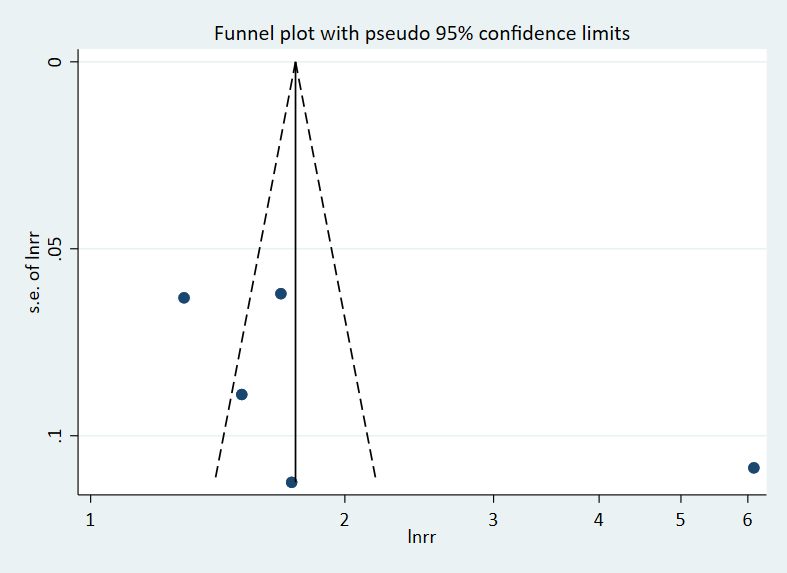


**Figure 3S. Funnel plot examining relationship between prelacteal feeds and any breastfeeding under six months.**


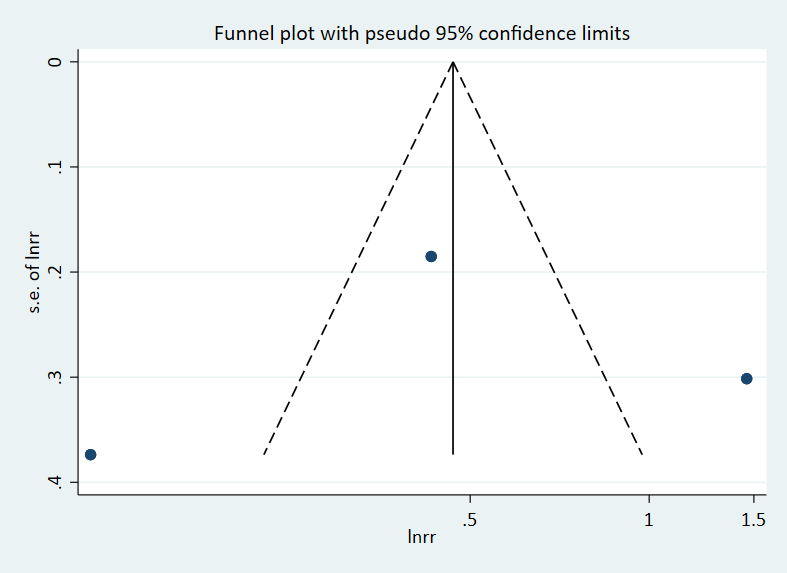


**Figure 4S. Funnel plot examining relationship between prelacteal feeds and any breastfeeding cessation up to one year.**


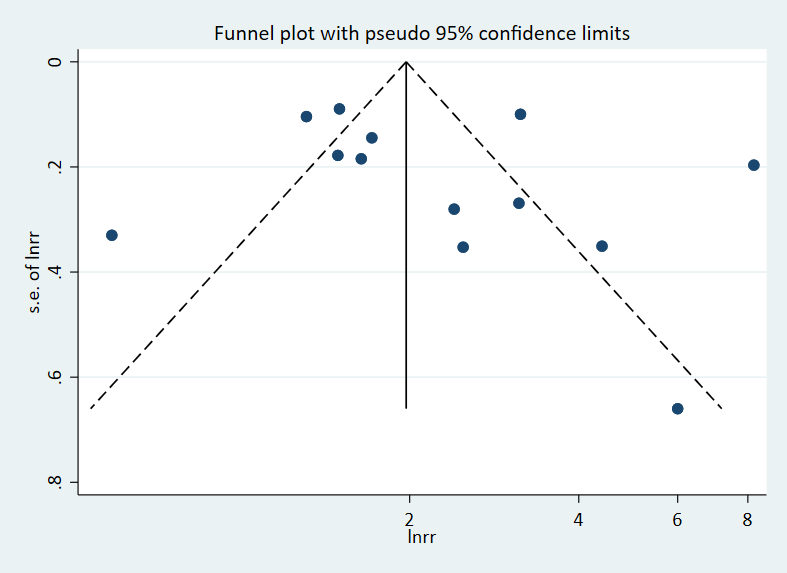


**Figure 5S. Funnel plot examining relationship between prelacteal feeds and exclusive breastfeeding under six months.**

**
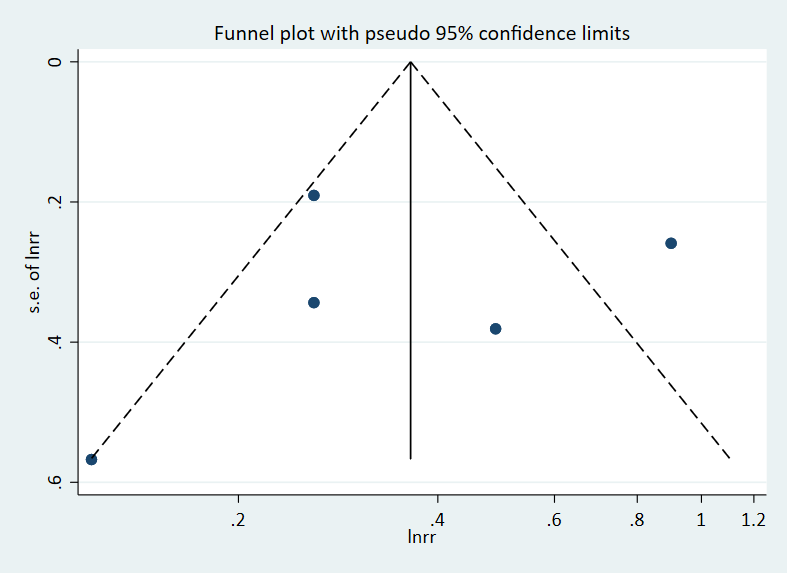
**
