# Supplementary material for: Impact of prelacteal feeds and neonatal introduction of breast milk substitutes on breastfeeding outcomes: A systematic review and meta‐analysis
Source: Matern Child Nutr. 2022 Apr 30;18(Suppl 3):e13368. doi: 10.1111/mcn.13368 (PMC9113480; doi:10.1111/mcn.13368)
Supplement: Supplementary file 6 — Supporting information. [file MCN-18-e13368-s004.docx]

| **Supplementary Table 1- Search history: The temporal relationship between prelacteals/early BMS introduction and breastfeeding failure** | |  |
| --- | --- | --- |
| Ovid MEDLINE(R) ALL <1946 to May 06, 2021> | |  |
| 1 | [infant feeding outcomes] | 0 |
| 2 | (breast fe* or breastfe*).mp. | 60518 |
| 3 | (formula or breastmilk substitute* or breast milk substitute*).mp. | 87622 |
| 4 | BMS.mp. | 7077 |
| 5 | mixed feeding.mp. | 543 |
| 6 | wean*.mp. | 54920 |
| 7 | infant feeding.mp. | 6214 |
| 8 | exp infant nutritional physiological phenomena/ | 60727 |
| 9 | milk substitutes/ or infant formula/ | 5117 |
| 10 | feed*.mp. | 592995 |
| 11 | or/2-10 | 737523 |
| 12 | ((after birth or "at birth" or post birth or postbirth) adj10 (formula or breastmilk substitute* or breast milk substitute* or BMS or supplementation or cows' milk or "other than breastmillk" or "other than breast milk")).mp. | 511 |
| 13 | (prelacteal* or pre-lacteal*).mp. | 324 |
| 14 | ((Formula supplementation adj5 hospital) or (Formula feeding adj5 hospital) or ("Formula use" adj5 hospital)).mp. | 64 |
| 15 | early supplementation.mp. | 153 |
| 16 | (in-hospital adj1 (formula or supplementation or "use")).mp. | 603 |
| 17 | early limited formula.mp. | 6 |
| 18 | (ihff or elf).mp. | 3486 |
| 19 | or/12-18 | 5090 |
| 20 | 11 and 19 | 886 |
| 21 | 20 not (animals not humans).sh. | 800 |
