# Supplementary material for: Impact of prelacteal feeds and neonatal introduction of breast milk substitutes on breastfeeding outcomes: A systematic review and meta‐analysis
Source: Matern Child Nutr. 2022 Apr 30;18(Suppl 3):e13368. doi: 10.1111/mcn.13368 (PMC9113480; doi:10.1111/mcn.13368)
Supplement: Supplementary file 7 — Supporting information. [file MCN-18-e13368-s005.docx]

**Supplementary Table 2**. Studies included and contribution to specific meta-analysis outcome.

| **Author** | **Year** | **Country** | **Design** | **Any BF under 6-months** | **Exclusive BF under 6-months** | **Any BF cessation at 1 y** | **Any BF cessation under 6-months** | **Exclusive BF cessation under 6-months** |
| --- | --- | --- | --- | --- | --- | --- | --- | --- |
| Alikasifoglu | 2001 | Turkey | Prospective cohort |  |  |  | x | x |
| Balogun | 2016 | Nigeria | Prospective cohort |  |  |  | x | x |
| Bruun | 2016 | Denmark | Prospective cohort |  |  |  | x |  |
| Forster | 2006 | Australia | Prospective cohort | x |  |  |  |  |
| Hossain | 1994 | Egypt | Prospective cohort |  | x |  |  |  |
| Patil | 2015 | Multi-country | Prospective cohort |  |  |  | x | x |
| Qiu | 2010 | China | Prospective cohort |  |  |  | x |  |
| Qiu | 2007 | China | Prospective cohort | x |  |  |  |  |
| Raheem | 2014 | Maldives | Prospective cohort |  |  |  | x |  |
| Richard | 2021 | Multi-country | Prospective cohort |  |  |  | x | x |
| Chantry | 2013 | United States | Prospective cohort |  |  |  | x |  |
| Dennis | 2019 | Canada | Prospective cohort |  | x |  |  |  |
| McCoy | 2020 | United States | Prospective cohort |  |  | x | x |  |
| Parry | 2013 | Hong Kong (China) | Prospective cohort |  |  | x |  |  |
| Semenic | 2008 | Canada | Prospective cohort |  |  |  |  | x |
| Sheehan | 1999 | Canada | Prospective cohort |  |  |  | x |  |
| Sheehan | 2006 | Canada | Prospective cohort |  |  |  | x |  |
| Tarrant | 2015 | Hong Kong (China) | Prospective cohort |  |  | x |  | x |
| Zakarija-Grkovic | 2016 | Croatia | Prospective cohort | x | x |  |  |  |
| Cardoso | 2010 | Chile | Prospective cohort |  |  |  |  | x |
| Zarshenas | 2020 | Iran | Prospective cohort |  |  |  | x |  |
| McDonald | 2010 | Australia | Prospective cohort |  |  | x |  | x |
| Agboado | 2010 | England | Prospective cohort |  |  |  | x |  |
| Dashti | 2014 | Kuwait | Prospective cohort |  |  |  | x |  |
| Raghavan | 2014 | India | Prospective cohort |  |  |  |  | x |
| Hruschka | 2003 | Guatemala | Prospective cohort |  |  |  | x |  |
| Dennis | 2014 | Canada | Prospective cohort |  | x |  |  |  |
