# Supplementary material for: Impact of prelacteal feeds and neonatal introduction of breast milk substitutes on breastfeeding outcomes: A systematic review and meta‐analysis
Source: Matern Child Nutr. 2022 Apr 30;18(Suppl 3):e13368. doi: 10.1111/mcn.13368 (PMC9113480; doi:10.1111/mcn.13368)
Supplement: Supplementary file 8 — Supporting information. [file MCN-18-e13368-s002.docx]

**Supplementary Table 3**. Studies not included and reasons for their exclusion in the meta-analysis.

| **Author** | **Year** | **Country** | **Design** | **Reasons for not inclusion** |
| --- | --- | --- | --- | --- |
| Hossain | 1992 | Egypt | Prospective cohort | Mean differences provided as outcome |
| Lakati | 2010 | Kenya | Prospective cohort | Confidence intervals not provided for the adjusted analysis |
| Rasheed | 2009 | Bangladesh | Prospective cohort | No group of analysis included not breastfed children for comparison |
| Demirci | 2017 | United States | Prospective cohort | Adjusted estimates not provided |
| Gray-Donald | 1985 | Canada | Clinital Trial | Only chi-squared tests used for statistical analysis |
| Hayek 2019 | 2019 | Isreal | Prospective cohort | Accelerated failure time provided as impact measure, and no Hazard Ratios |
| McKinney | 2016 | United States | Prospective cohort | Impact measures reported as beta coefficients. Moreover, racial disparities were the main exposures, while BMS/prelacteal were considered as mediators |
| Samuels | 1985 | United States | Prospective cohort | No impact measures along with 95% CI provided |
| Sheehan | 1999 | Australia | Quase-exp design | No impact measures along with 95% CI provided |
| Feinstein | 1986 | United States | Prospective cohort | No impact measures along with 95% CI provided |
| Vehling | 2018 | Canada | Prospective cohort | The only study that children were followed-up up to 2 years |
| Weisband | 2017 | United States | Prospective cohort | Linear regression models used |
| Hossain | 1995 | Egypt | Prospective cohort | Ourcome was timing of breastfeeding intiation |
